# The Potential Benefits of Non-skills Training (Mental Toughness) for Elite Athletes: Coping With the Negative Psychological Effects of the COVID-19 Pandemic

**DOI:** 10.3389/fspor.2021.581431

**Published:** 2021-09-24

**Authors:** Neil Dagnall, Kenneth Graham Drinkwater, Andrew Denovan, R. Stephen Walsh

**Affiliations:** Department of Psychology, Manchester Metropolitan University, Manchester, United Kingdom

**Keywords:** mental toughness, COVID-19, non-cognitive skills, elite athletes, positive psychology

## Abstract

The spread of COVID-19 has had a significant impact on global sport. This is especially true at the elite level, where it has disrupted training and competition. Concomitantly, restrictions have disrupted long-term event planning. Many elite athletes remain unsure when major events will occur and worry about further interruptions. Although some athletes have successfully adapted to the demands of the COVID-19 crisis, many have experienced difficulties adjusting. This has resulted in psychological complications including increased stress, anxiety, and depression. This article critically examines the extent to which non-cognitive skills training, in the form of increased awareness of Mental Toughness, can help elite athletes inoculate against and cope with negative psychological effects arising from the COVID-19 pandemic. Non-cognitive skills encompass intrapersonal (motivations, learning strategies, and self-regulation) and interpersonal (interactions with others) domains not directly affected by intellectual capacity. Previous research indicates that enhancement of these spheres can assist performance and enhance mental well-being. Moreover, it suggests that training in the form of increased awareness of Mental Toughness, can improve the ability to cope with COVID-19 related challenges. In this context, Mental Toughness encompasses a broad set of enabling attributes (i.e., inherent and evolved values, attitudes, emotions, and cognitions). Indeed, academics commonly regard Mental Toughness as a resistance resource that protects against stress. Accordingly, this article advocates the use of the 4/6Cs model of Mental Toughness (i.e., Challenge, Commitment, Control, and Confidence) to counter negative psychological effects arising from COVID-19.

## Introduction

This article outlines how Mental Toughness training can help elite athletes cope with negative psychological effects arising from the COVID-19 pandemic. The spread of COVID-19 has had a significant impact on global sport. This is especially true at the elite level, where it has interrupted regular competition (e.g., The Super League), lead to rescheduling (e.g., Deontay Wilder vs. Tyson Fury III), and disrupted routine training (World Health Organization, 2020). In addition, ensuing COVID-19 restrictions have affected long-term schedules and major event planning (e.g., the 2020 UEFA European Football Championship, and both the Tokyo 2020 Summer Olympics and Paralympics). Accordingly, many elite athletes remain unsure when competitions will occur, and worry about potential further delays. Although some athletes have successfully adapted to the demands of the COVID-19 crisis, many have experienced difficulties adjusting. Noting this, sporting bodies have become concerned about the psychological well-being of elite performers (Mehrsafar et al., 2020; Ivarsson et al., 2021).

Professional athletes are particularly vulnerable to the negative effects of COVID-19 disruptions because sport is the focus of their lives. It represents a core component of self-identity, is the sportspersons' vocation, and principal source of financial income. For these reasons, disturbance(s) to sporting schedules can cause uncertainty, prove unsettling, and in extreme cases produce psychological distress. This notion is consistent with the observation that increased levels of stress, depression and anxiety are frequently reported psychological responses to the COVID-19 crisis (Rajkumar, 2020; Mojtahedi et al., 2021).

In the case of sportspersons, negative psychological effects can manifest in a variety of ways. Pillay et al. (2020), who surveyed athletes in the final phase of the South African lockdown, found that many experienced feelings of depression (52%). Similarly, a significant number required motivation to keep active (55%), consumed excessive carbohydrates (76%), and reported altered sleep patterns (79%). These findings align with studies that predicted an increased demand for psychological services (Toresdahl and Asif, 2020; see Schinke et al., 2020; i.e., sport psychologists and counselors). A common issue being the inability to manage effectively COVID-19 related stress. Specific concerns centered on fear of infection, recovery from contagion, disruption to training, maintaining diet, changes in activity levels, inability to access training facilities, social isolation, disturbed sleep, and domestic complications (Kizhakkekara et al., 2021).

Concurrently, athletes often experience performance anxieties including apprehensions about the effect of interruptions on their ability to return to competition standard and sustain peak levels. This concurs with the notion that stress is detrimental when it arises from events that threaten the individual's sense of adaptive adequacy. Particularly when ensuing demands exceed the perceived capacity to cope and (Sherman and Cohen, 2006; Thelwell et al., 2017).

Noting these issues, Jukic et al. (2020) suggest that it is useful to encourage athletes to retune their mindset, so that COVID-19 disruptions are contextualized as a “positive” opportunity for personal development. Perceiving change as a constructive space for professional reflection and individual growth, potentially reduces negative psychological effects such as mental fatigue. This is especially true if support structures exist in the form of education (preventive behavior, hygiene measures, and recovery), daily conditioning advice, frequent monitoring, and psychological training (i.e., meditation, mindfulness, and deep breathing exercises), and individual athletes feel empowered to exploit them.

Regarding psychological training, sportspersons who effectively utilize available cognitive resources and successfully implement coping strategies will be best equipped to manage attendant mental distress (Jukic et al., 2020). However, not all athletes possess sufficient understanding of psychological processes to successfully manage their well-being. Recognizing this, the present article examines ways in which non-cognitive skills training, in the form of increased knowledge of Mental Toughness, can facilitate the development of self-efficacy and inoculate/moderate the negative psychological impact of COVID-19.

## Non-cognitive Skills and Mental Toughness Training

Non-cognitive skills denote abilities not directly affected by intellectual capacity. Although, this term is a misnomer to the extent that performance inherently relies upon cognitive processing (Borghans et al., 2008), researchers use the label to designate abilities or skills beyond those assessed by standard intelligence tests. Thus, non-cognitive skills encompass intrapersonal (motivations, learning strategies, and self-regulation) and interpersonal (interactions with others) domains. Previous research indicates that enhancement of non-cognitive skills across a range of applied spheres (i.e., sport, vocational, and educational) can aid performance (Lin et al., 2017), and suggests that non-cognitive skills training will improve the ability to cope with the challenges of COVID-19.

This is especially true of Mental Toughness, which is associated with positive psychological outcomes including increased psychological well-being and enhanced flow (i.e., the tendency to experience a psychological state of optimal experience) in real-world settings (Jackman et al., 2016). Pertinent to the present article, researchers have found that this is especially true in the context of sport (Golby and Wood, 2016). Moreover, the importance of Mental Toughness in optimizing performance is widely acknowledged within athletic settings (coaches, sports psychologists, athletes, etc.) (Goldberg, 1998; Stamatis et al., 2020). This perspective is commensurate with the delineation of Mental Toughness as an important factor in fostering adaptive responses to positively and negatively construed pressures, situations, and events (Cowden, 2017, Lin et al., 2017).

The contemporary conceptualization of Mental Toughness derives from work on hardiness (Maddi, 2002). Hardiness comprises three factors: Commitment (deep involvement in life activities), Challenge (expectation that life is unpredictable and that changes stimulate personal development), and Control (the desire to influence outcomes) (Kobasa, 1979; Maddi, 2002). Noting that hardiness protected against psychological distress but failed to account for the unique demands of competitive sport, Clough et al. (2002) added a Confidence dimension to form their Mental Toughness model (Sheard, 2012). This modification was consistent with the observation that self-belief is a key component of competitive success (Lane, 2014).

For these reasons, development of Mental Toughness can help to counter the negative psychological effects of the COVID-19 pandemic. The potential effectiveness of Mental Toughness arises from the fact that the construct encompasses a broad set of attributes (i.e., inherent and evolved values, attitudes, emotions, and cognitions). These not only aid goal achievement, but concomitantly influence the way people approach, react to, and evaluate pressure, challenge, and difficulty. Thus, Mental Toughness is commonly viewed as a resistance resource linked to coping (Nicholls et al., 2011). Relevant to the present article, this abstraction is consistent with the original delineation of Mental Toughness. This derived from work with elite athletes and viewed Mental Toughness as synonymous with stress tolerance and maximized performance (Loehr, 1982, 1986, 1994; Earle, 2006).

Consistent with this view, Gerber et al. (2013b) found that higher levels of Mental Toughness within high school and undergraduate students were associated with reduced levels of depressive symptoms arising from high-stress situations. Moreover, high levels of Mental Toughness predict stress resilience, ward off depression, and help to maintain life satisfaction (Gerber et al., 2013a). Other studies also link high levels of Mental Toughness to health benefits (i.e., improved sleep quality; Brand et al., 2014). Noting this, theorists have recently framed Mental Toughness as a range of psychological resources that promote positive mental health (Lin et al., 2017; Drinkwater et al., 2019; Papageorgiou et al., 2019a,b). The positive effects of Mental Toughness are well documented in sport. For instance, Gucciardi and Jones (2012) showed small to moderate negative correlations between Mental Toughness and stress, anxiety, and depression in cricketers.

The benefits of Mental Toughness may arise from related coping strategies. This was demonstrated by Nicholls et al. (2008), who reported in a large sample of athletes that higher levels of Mental Toughness correlated with employment of more problem or approach-oriented strategies (i.e., logical analysis, thought control effort expenditure, and mental imagery) and less use of avoidance (resignation, mental distraction, and distancing). This finding concurs with the view that mental toughness in athletes can moderate negative consequences resulting from exposure to high stress (Gerber et al., 2018).

Observing conceptual difficulties with the definition of mental toughness, Gucciardi redefined the construct as “a state-like psychological resource that is purposeful, flexible, and efficient in nature for the enactment and maintenance of goal-directed pursuits” (p. 18, Gucciardi, 2017). This delineation acknowledges the roots of Mental Toughness and its use in applied settings, such as dealing with the negative psychological effects of the COVID-19 pandemic.

## The 4/6Cs Model of Mental Toughness

The multidimensional framework proposed by Clough et al. (2002) could provide vital insights into the negative psychological effects that the COVID-19 pandemic has had on elite athletes. This encompasses four factors: Commitment, Challenge, Control, and Confidence. Commitment, also referred to as “stickability,” is the ability to carry out tasks successfully, despite problems/obstacles. Challenge denotes the extent to which individuals view change, setbacks, and tests as opportunities. Control represents the perception that individuals feel able to manipulate their environment. This dimension is subdivided into Emotional (the facility to keep anxieties in check) and Life (capacity to realize plans and make a difference). Confidence indexes self-belief in Abilities (individual worth) and Interpersonal capabilities (less likely to be intimidated in social settings). Acknowledging these subdivisions some researchers also call the 4Cs, the 6Cs (Poulus et al., 2020).

The 4/6Cs are encapsulated within the 48-item Mental Toughness Questionnaire (MTQ48; Clough et al., 2002), which produces both total and dimensional scores (see Perry et al., 2021). Although there has been debate about the stability of the model (see Gucciardi et al., 2012, 2013), Clough et al. (Perry et al., 2013, 2015, 2021) and independent researchers (e.g., Horsburgh et al., 2009) have published articles that demonstrate the model's factorial validity. Consequently, the MTQ48 is a generally accepted, widely used measure of Mental Toughness (Dagnall et al., 2019).

The strength of the 4/6Cs model, when applied to assessing the negative psychological effects of COVID-19, is that it can reveal individual areas of mental sensitivity. Indeed, as a commercial tool the instrument provides feedback specific to each of the 4/6Cs. This information is potentially useful for the development of individual coping strategies.

## The MTQ48 and the Negative Psychological Effects of Covid-19

The strength of the 4/6Cs Model is that it can be used to signpost specific, personal areas of stress susceptibility (values, attitudes, emotions, and cognitions). This is useful for appreciating the psychological consequences that the COVID-19 crisis has had on an individual, and for identifying at risk sportspersons. Knowledge of undesirable mental impact and discrete vulnerability can inform the development of specialized personal coping strategies. Explicitly, interventions that recognize personal sensitivities, robustness (“toughness”), and the uniqueness of each sportspersons' psychological profile. This approach is conceptually sound, since previous research has established that higher levels of Mental Toughness are associated with positive outcomes across a range of applied settings (sport: Meggs et al., 2019; occupational: Marchant et al., 2009; educational: St Clair-Thompson et al., 2015; and health: Kruger, 2018). In this context, the 4/6Cs Model (Clough et al., 2002) can act as a nuanced tool for detecting both vulnerability to, and consequences of the negative psychological effects of the COVID-19 pandemic on elite athletes (Clough et al., 2016).

Applied to sportspersons, the 4/6Cs model can make specific predictions regarding the types of difficulties individuals are likely to experience as a function of lower scores on each of the Mental Toughness dimensions (Clough and Strycharczyk, 2012). Athletes scoring low on Commitment will be prone to distractions and accordingly find it difficult to complete tasks including exercise/training routines. Moreover, they may lack perseverance when confronted by sustained barriers (i.e., extended lockdown). Similarly, sportspersons scoring low on Challenge may become psychologically overwhelmed by difficulties arising from COVID-19. Particularly instability and unpredictability will prove uncomfortable, and tax the ability to cope. This can manifest as a tendency to focus on the detrimental consequences of enforced changes (e.g., postponement of competition and disruption to training), and a slowness to adapt. Potentially, resulting in a failure to realize developmental opportunities. Over time, athletes low in Challenge will perceive sustained pressure as wearisome and become risk aversive. This may mean they become unwilling to explore alternative fitness and practice exercises.

Athletes scoring low on Confidence generally lack self-belief. During the COVID-19 crisis this is likely expressed as increased self-criticism and/or despondence. This can manifest as an overreliance on others, and a reluctance to assume responsibility/show initiative. In terms of Ability, low self-assurance often presents as concerns about capability, excessive worry, and the tendency to underestimate skills, knowledge, abilities, and importance. Interpersonally, lack of confidence often results in disengagement from the training group or team. This can lead to psychological distancing and the self-perception that the individual is not generally valued. Low Interpersonal Confidence is characterized by exaggerated concern for others' views and opinions. COVID-19 related isolation and distancing is likely to amplify these perceptions.

Finally, low levels of Control indicate perceived lack of volition, autonomy, and impact. In relation to the COVID-19 crisis, this can result in a sense of powerlessness. Accordingly, sportspersons are likely to feel they are unable to meaningfully influence factors. Relatedly, they may apply themselves to tasks haphazardly; devoting unnecessary effort and resources to aspects outside of their governance, and too quickly withdraw from matters they could influence. In terms of Emotional Control, low scores often reflect high levels of anxiety and a lack of affective adaptation. This is typically accompanied by overt expression of feelings. In terms of Life Control, low scores are characterized by perceptions of ineffectiveness and unimportance. At the problem-solving level, this hinders multi-tasking and is typified by the tendency to be easily defeated by setbacks.

Awareness of Mental Toughness and the 4/6Cs is important because it can inform the development of strategies to negate (inoculate) negative psychological effects, or it can suggest coping mechanisms to alleviate problems arising from the COVID-19 crisis (i.e., stress, anxiety, and depression). In both instances, knowledge of Mental Toughness and its psychological benefits can be helpful to elite athletes who are experiencing difficulties acclimatizing to the “new” normal. This approach has been highly successful across a range of applied settings (see Lin et al., 2017). This suggests that athletes would benefit from greater awareness of Mental Toughness generally, and the use of strategies targeted to enhance personal areas of sensitivity that are likely to influence outlook and subsequent performance.

These could utilize specific methods to enhance sensitive 4/6C dimensions. Particularly, coaches could increase levels of Commitment by encouraging athletes to focus on their enjoyment/love of the sport and achievable goal setting (Weinberg et al., 2001; Leyton-Román et al., 2021). Established approaches to enhancing Challenge include the identification of self-referenced standards, use of targeted objectives, and rivalries with other athletes (Crust and Clough, 2011). Regarding both Confidence in abilities (individual worth) and Interpersonal capabilities, coaches could facilitate these factors by encouraging individuals to be appropriately assertive and expressive in performance and everyday life (Thelwell et al., 2017). For instance, develop a sense of self and presence related to role and performance.

Finally, trainers can develop Life Control within sportspersons through the referencing of previous achievements and accomplishments and imagining optimal performance (Erdner and Wright, 2018). These interventions are likely to be associated with heightened self-efficacy, the individual's belief in their ability to succeed in specific situations (Munroe-Chandler et al., 2008). Regarding Emotional Control, coaches can improve this factor by developing a sportsperson's understanding of emotion regulation strategies (i.e., cognitive reappraisal, distraction, and action control). Familiarity with these concepts is importance since adaptive emotional control requires the ability to flexibly switch between emotion control strategies (Koch et al., 2018).

## Discussion

The application of the 4/6C Model to elite athletes will provide researchers with a nuanced understanding of the negative effects of the COVID-19 pandemic at both a general and personal level. At an individual level, MTQ48 feedback serves as a tool for signposting psychological areas of stress vulnerability (sensitivity) and probable adverse impacts (i.e., problematic values, attitudes, emotions, and cognitions). This information can play an important role in both preventing and identifying mental distress. Recognizing detrimental states can inform the advancement of coping strategies that can protect against/decrease the influence of negative psychological states arising from COVID-19.

Moreover, the 4/6C Model offers researchers a conceptual framework for designing person-centered, targeted interventions (Clough et al., 2016; Zalewska et al., 2019). In this context, the breadth of the model allows for the integration of a range of psychological and cognitive techniques tailored to the needs of a sportsperson. This individualized approach is consistent with the observation that therapeutic advice is best received when it is perceived as pertinent, collaborative, and helpful (Bachelor, 2013).

The success of the proposed application rests on the assumptions that Mental Toughness is malleable, and that increases in Mental Toughness resulting from psychological training concurrently enhance positive psychological elements. There is significant evidence to support these propositions (see Lin et al., 2017). The view that Mental Toughness is adaptable aligns with the elucidation of the construct as a “plastic” trait that can develop over time (Strycharczyk and Clough, 2014), and concurs with behavioral genetic studies that find around half of the variance in the construct is attributable to non-shared environmental factors (e.g., Horsburgh et al., 2009; Veselka et al., 2009). Further consideration of this evidence, however, suggests an important training consideration. Explicitly, that improvements in mental toughness are best achieved by focusing on the dimensions with the lowest heritability (i.e., Commitment and Control).

Moreover, Gucciardi et al. (2009a,b) observed that interventions in the form of psychological skills training (PST) programs increased athletes' ratings of Mental Toughness. Similarly, Sheard and Golby (2006) found that PST increased Mental Toughness, promoted psychological development, and aided the performance of adolescent swimmers. Extending this to consider whether PST enhanced positive psychological characteristics, Golby and Wood (2016) reported that PST increased levels of Mental Toughness and concomitantly heightened levels of perceived self-efficacy, positive affect, and self-esteem in student-athlete rowers. This supports the notions that Mental Toughness is trainable and that interventions can facilitate psychological well-being.

The use of Mental Toughness interventions requires cautious development and implementation for several reasons. Firstly, it is difficult to generalize across previous studies because they have often used small, highly specialized samples (e.g., Bull et al., 2005; Wilson et al., 2019). This makes comparisons difficult due to inherent variations in potentially conflating factors involving level of performance, sport, age, etc. Furthermore, although Mental Toughness studies frequently focus on “elite athletes” and “performers” these terms do not denote a homogeneous group. Rather, they indicate that individuals in a particular sport have reached a relatively high standard within that domain. Scrutiny reveals the presence of important differences in competition standard that may influence the effectiveness of interventions. Thus, although much work has used common respondent selection criteria (e.g., Woodman and Hardy, 2001; Hanton and Connaughton, 2002), such as full international honors and represented their country in major events (e.g., Olympic Games) (Jones, 2002), researchers have frequently employed different prescriptions (e.g., Cowden et al., 2014, tennis players; Bull et al., 2005, English cricketers).

In addition, papers have derived conclusions on the effectiveness of Mental Toughness using various methodological approaches (i.e., self-report, interview, and focus group) and data analytical techniques. Valid suppositions are only likely to arise from systematic triangulation of prior research. Less rigorous evaluations are likely to result in superficial inferences. A further limitation to consider is that preceding work has employed a range of different indices of Mental Toughness. These have different factorial structures and vary in their conceptualization of Mental Toughness. For instance, The Sport Mental Toughness Questionnaire (SMTQ, Sheard et al., 2009) yields a general score derived from Confidence, Constancy, and Control. Clearly, personalized interventions based on SMTQ scores are likely to deviate from those predicated on the MTQ48.

Related to the use of self-report measures, much of the extant work on interventions has employed cross-sectional designs, with data collection occurring at one point in time. This approach focuses on relationships between variables and cannot establish causations (Nicholls et al., 2008). To establish causality or directionality researchers need to use experimental manipulation or assess changes in scores across multiple time points.

Noting these factors, while the body of research indicates that interventions can increase Mental Toughness and concomitantly have adaptive psychological benefits (i.e., facilitate coping), researchers and practitioners should carefully interrogate studies from which these conclusions derive before designing and administering interventions. Likewise, they should cautiously pilot interventions, carefully monitor and document outcomes (Stamatis et al., 2020).

A further caveat is that individuals high in Mental Toughness often exhibit socially malevolent characteristics (e.g., ruthless, and selfish), especially when they are striving to achieve goals (Sabouri et al., 2016). Hence, elements of Mental Toughness, particularly those associated with dark triad traits (i.e., narcissism, psychopathy, and Machiavellianism), may have detrimental effects on psychological health (Golby and Wood, 2016). Although, research in this area is relatively underdeveloped, recent evidence indicates that this supposition is overly simplistic because relationships between Mental Toughness, dark triad traits and psychological well-being are highly complex.

For example, Papageorgiou et al. (2019a) found that Mental Toughness mediated the effects of Subclinical Narcissism (SN) on depression, resulting in lower levels of depression. Moreover, Papageorgiou et al. (2019a) reported that SN increases Mental Toughness and reduces perceived stress. Exploring the link between SN with prosocial traits can be particularly helpful when seeking to identify and promote its adaptive tendencies against symptoms of psychopathology. As this is a nascent area of work, future research should further examine relationships between Mental Toughness, dark triad traits and psychological well-being. Also, to maximize the therapeutic use of Mental Toughness, subsequent work should seek to identify non-adaptive elements.

It is important to acknowledge that not all academics would support the proposed application of the 4/6C Model. Opponents argue that its usefulness is limited by conceptual deficiencies (Gucciardi, 2017). Frequently cited criticisms center on the insistence that Mental Toughness is a unidimensional rather than multidimensional construct, variations in factor structure as a function of context, observed deficiencies in measurement properties, and the authors' failure to specify item selection processes (Gucciardi et al., 2012, 2013; Gucciardi, 2017).

Noting these concerns advocates of the 4/6Cs Model have robustly defended their position (e.g., Clough et al., 2012). Commensurate with this stance, the MTQ48 remains a widely used, psychometrically validated instrument, which is supported by robust empirical evidence. Moreover, while different definitions and abstractions of Mental Toughness exist, delimitations share core characteristics (i.e., self-belief, persistence on achieving goals, motivation, and the ability to deal with setbacks). Indeed, these are integral elements of the 4/6Cs model. Further support for the applicability of the 4/6Cs to therapeutic contexts is provided by a wealth of previous related research (see review article of Lin et al., 2017).

## Data Availability Statement

The original contributions presented in the study are included in the article/supplementary material, further inquiries can be directed to the corresponding author.

## Author Contributions

ND collated ideas and organized content. KD assisted with drafting. AD advised on content. RW provided feedback on drafts. All authors contributed to the production of the final draft.

## Conflict of Interest

The authors declare that the research was conducted in the absence of any commercial or financial relationships that could be construed as a potential conflict of interest.

## Publisher's Note

All claims expressed in this article are solely those of the authors and do not necessarily represent those of their affiliated organizations, or those of the publisher, the editors and the reviewers. Any product that may be evaluated in this article, or claim that may be made by its manufacturer, is not guaranteed or endorsed by the publisher.
